# Immunomodulatory drug discovery from herbal medicines: Insights from organ-specific activity and xenobiotic defenses

**DOI:** 10.7554/eLife.73673

**Published:** 2021-11-15

**Authors:** Jue Shi, Jui-Hsia Weng, Timothy J Mitchison

**Affiliations:** 1 Centre for Quantitative Systems Biology, Department of Physics and Department of Biology, Hong Kong Baptist University Hong Kong China; 2 Department of Systems Biology, Harvard Medical School Boston United States; 3 Institute of Biological Chemistry, Academia Sinica Taipei Taiwan; Harvard Medical School United States; Harvard Medical School United States

**Keywords:** immunomodulatory drug, xenobiotic defense, drug discovery, plant-derived molecules, traditional chinese medicine, organ-specific drug action

## Abstract

Traditional herbal medicines, which emphasize a holistic, patient-centric view of disease treatment, provide an exciting starting point for discovery of new immunomodulatory drugs. Progress on identification of herbal molecules with proven single agent activity has been slow, in part because of insufficient consideration of pharmacology fundamentals. Many molecules derived from medicinal plants exhibit low oral bioavailability and rapid clearance, leading to low systemic exposure. Recent research suggests that such molecules can act locally in the gut or liver to activate xenobiotic defense pathways that trigger beneficial systemic effects on the immune system. We discuss this hypothesis in the context of four plant-derived molecules with immunomodulatory activity: indigo, polysaccharides, colchicine, and ginsenosides. We end by proposing research strategies for identification of novel immunomodulatory drugs from herbal medicine sources that are informed by the possibility of local action in the gut or liver, leading to generation of systemic immune mediators.

## Introduction

There are strong demands to develop new immunomodulatory drugs to combat chronic diseases. In the broadest sense, we need to inhibit specific arms of the immune system in inflammatory diseases, neurodegeneration and hematological cancers, or enhance them in solid cancers. Most current efforts aim at biologic drugs (antibodies, cytokines, decoy receptors etc.) that are highly specific for extracellular targets, mostly cytokines and their receptors. Biologics are considered faster and less risky to develop than small-molecule drugs, and biologic antagonists of cytokines and paracrine signals are blockbuster medicines in inflammation and cancer. However, small molecules still offer important benefits. Notably, they are much easier to manufacture and more affordable once they come off patent, which suits global needs. One small-molecule class, kinase inhibitors, has been extensively explored for immunomodulation, and inhibitors of JAK, BTK, and SYK kinases have been approved to downregulate specific immune pathways in various diseases (Zarrin et al., 2021). There is also great interest in adding small molecules in combination to help patient subgroups that are insufficiently responsive to single biologics (Adams et al., 2015; van der Zanden et al., 2020; Ahmed et al., 2021).

Here, we discuss the potential of Traditional Chinese Medicines (TCM) and other herbal medicine traditions as starting points for new immunomodulatory drugs from a specific perspective: that plant-derived molecules may have low oral bioavailability and restricted distribution that lead to organ-specific pharmacology. In standard oral pharmacology models, drugs are efficiently adsorbed into the bloodstream and then act throughout the body via transport in blood. This standard model is often assumed for active molecules present in traditional medicines. We will argue that for some TCM molecules, their chemical nature and the xenobiotic defense systems in the human body point to an alternative model, where the drug acts locally to induce circulating mediators with systemic immunomodulatory activity.

In this review we will not address two important areas in TCM pharmacology, combinatorial drug action and modulation of the gut microbiome. Combining ingredients with complementary actions is central to the TCM philosophy (Zhang et al., 2014), which prompted the application of systems pharmacology approaches to rationally decipher existing combinations and develop new ones (Li et al., 2012; Ru et al., 2014). The physical emulsions naturally present in herbal extracts may also increase oral bioavailability of active components compared to pure molecules (Zhao et al., 2020). However, drug approval in the West usually requires a pure molecule with a precisely defined formulation to demonstrate single agent activity before combinations can be tested, and we are convinced that many TCM molecules with useful single agent activity remain to be discovered. Modulation of the gut microbiome by TCMs is an important research frontier (Lin et al., 2019b; Lin et al., 2021; Zhang et al., 2020a; Li et al., 2021b). Here, we will only touch on this topic in the context of immune regulators that act locally in the gut.

### Xenobiotic defenses and their role in TCM pharmacology

Traditional TCM prescriptions mainly consist of dried plant and fungal ingredients boiled in water and administered as oral decoctions. Thus, consideration of oral bioavailability is paramount in discussing their mechanisms and pharmacology. Western drugs designed for oral dosing are usually optimized using synthetic chemistry and formulation methodology to maximize adsorption through the gut wall and minimize metabolism by the liver. This leads to drugs with high oral bioavailability, long plasma half-life and high systemic exposure. A drug with these properties is said to display ‘good pharmacology’. Some famous plant-derived drugs display good pharmacology, such as morphine, digoxin, quinine and artemisinin. However, most TCM molecules display ‘poor pharmacology’, that is poor oral bioavailability, short plasma half-life and rapid metabolism by the gut and liver (Lin et al., 2019b; Chen et al., 2016). How molecules with these properties can achieve beneficial systemic action is a long-standing puzzle in TCM pharmacology. The gut microbiome may play a role in mediating systemic actions of some oral TCM components that are poorly absorbed (Chen et al., 2016). We will focus on an alternative model, that local action in the gut or liver generates systemic mediators.

The “poor pharmacology” of most TCM molecules is not a coincidence. Rather, it results from evolutionary fundamentals. Plants and fungi evolved complex, bioactive metabolites, which we will term “xenobiotics”, mainly to deter herbivores through bitter taste or/and poisonous actions. In parallel, animals evolved xenobiotic defenses to protect them from potential toxins in their diets (Florsheim et al., 2021). Animals that could safely digest toxic plants gained a competitive advantage in access to food resources. Xenobiotic defense systems evolved to protect animals against precisely the kinds of molecules present in TCM oral decoctions, which is why, in our view, most TCM molecules exhibit poor pharmacology. Here, we will argue that the detection-response arm of xenobiotic defenses may also help explain their therapeutic actions.

The first layer of xenobiotic defenses in animals are sensory cues, such as bitter taste or bright colors, which signal avoidance of potentially poisonous foods. The second is a series of barriers that prevent systemic exposure, of which the gut wall is the most important. The third is a complex series of detection, transport and metabolism systems which rapidly detoxify xenobiotics that enter the bloodstream and trigger protective gene expression in exposed organs. The latter are concentrated in the liver in humans and, to a lesser extent, in the kidney. The liver efficiently removes many xenobiotics from the portal circulation before they enter the systemic circulation. This is achieved by its filter-like anatomy, drug transporters expressed on the sinusoidal (blood facing) membrane of hepatocytes and enzymes that rapidly metabolize xenobiotics into derivatives that are less toxic and easier to be excreted. This filtering effect of the liver, which can be upregulated by prior exposure to xenobiotics, is called ‘first pass metabolism’ and is responsible for the poor pharmacology of many plant-derived xenobiotics that cross the gut barrier.

Importantly, the xenobiotic defense systems of the gut, liver and kidney are dynamic. Maintaining high levels of defense is metabolically costly, therefore these systems evolved to be inducible. Exposure to xenobiotics induces defense proteins, notably metabolic enzymes and transporters, at the transcriptional level in the gut, liver and other organs (Xie et al., 2004; Müller, 2000). Xenobiotics are detected in part by their toxic actions on cellular organelles and in part by dedicated receptor proteins which are thought to have evolved for xenobiotic sensing (Negishi et al., 2020; Hoffmann and Partridge, 2015). Examples of xenobiotic sensors that are prominent in hepatocytes include the nuclear receptors PXR (Pregnane X receptor), FXR (Farnesoid X receptor) and AHR (Aryl Hydrocarbon Receptor). These receptors bind xenobiotics through the ligand-binding domains, leading to allosteric activation of their gene expression domains (Mackowiak and Wang, 2016). The chaperone-like sensor KEAP1 (Kelch Like ECH Associated Protein 1) detects oxidizing and alkylating xenobiotics using thiol chemistry. Oxidation and alkylation of cysteines on KEAP1 cause it to be degraded, which releases and activates its client protein, the transcription factor NRF2 (Baird and Yamamoto, 2020). NRF2 then serves as a master activator of anti-oxidant and xenobiotic defenses. Activation of xenobiotic sensors in hepatocytes leads to induction of drug metabolizing enzymes, such as cytochrome P450s, as well as efflux pumps, anti-oxidant proteins and other defense factors. Induction of xenobiotic defenses, especially P450s, is an important cause of drug-drug interactions (Ma, 2008). This is one reason patients must inform their doctors of all the drugs they are taking, including herbal supplements and traditional medicines. Induction of xenobiotic defenses by drugs has been most studied in the liver, but it also occurs in the gut and other tissues (Dressman and Thelen, 2009; Stresser et al., 2021).

The most important cell type for detecting and responding to xenobiotics is the hepatocyte. Research on xenobiotic sensors and the protective genes that they regulate has focused on two functions that are local to hepatocytes, that is, regulation of drug metabolism (Guengerich, 2017) and protection from the cytopathic effects of toxins, particularly by the KEAP1-NRF2 system (Taguchi and Kensler, 2020). Their roles in regulating inflammatory pathways and promoting whole-organism homeostasis have been much less studied. Of particular importance to this review is that activation of xenobiotic sensors in the gut or liver can induce secreted proteins, whose effects extend to the whole body. Signaling proteins secreted by the gut or liver are termed, respectively, enterokines and hepatokines. A systematic analysis of drug- and toxin-induced changes in rat liver gene expression revealed that many xenobiotics induce or repress hepatokines with known systemic actions, including IGF1, IGFALS, IGFBP, and GDF15 (Shimada and Mitchison, 2019). This regulation implies that xenobiotic sensors in the liver can mobilize the whole human body to regulate feeding, metabolism and other physiological processes. The same is likely true of the gut, although this has been less studied. Activation of xenobiotic defenses has been implicated in healthy aging of entire organisms (Hoffmann and Partridge, 2015). Here, we will link it to the action of herbal medicines. Xenobiotic sensors also monitor gut microbiome metabolites (Stockinger et al., 2021), so they are a point of integration between direct- and microbiome-mediated effects of small molecules on the human body.

The innate immune system can detect and respond to xenobiotics and we believe this system plays an important role in immunomodulation by herbal medicines, especially for molecules that lack oral bioavailability and act locally in the gut. This is a newer area of research compared to xenobiotic defenses in hepatocytes and much remains to be learned. Macrophages and dendritic cells in the lamina propria continuously sample the gut luminal contents by extending phagocytic processes directly into the gut lumen (‘periscoping’) and also by receiving material that is transcytosed out of the lumen by Microfold cells (M cells) (Bain and Schridde, 2018; Strober, 2009). This sampling is important for monitoring the microbiome and triggering immune responses in the gut. We propose it also constitutes a path by which insoluble xenobiotics that are unable to cross the gut wall due to their particulate form can access the immune system. Xenobiotic receptors in gut macrophages that have been implicated in sensing both microbiome metabolites and xenobiotics include AHR (Stockinger et al., 2021) and Toll-like receptors (TLRs) (Abreu, 2010). TLRs are usually thought of as receptors for pathogen-derived molecules, but as we will discuss below, they can also bind TCM polysaccharides. Modulation of gut macrophages and dendritic cells by luminal TCM molecules is expected to modulate inflammation locally, which may be important in treatment of gut inflammatory diseases, as discussed below. It can also give rise to systemic effects in at least two ways, by triggering secretion of circulating cytokines and by trafficking of educated leukocytes from the gut to other organs. We speculate both are important for relaying the effects of xenobiotic exposure from the gut to distant sites.

**Figure 1.**
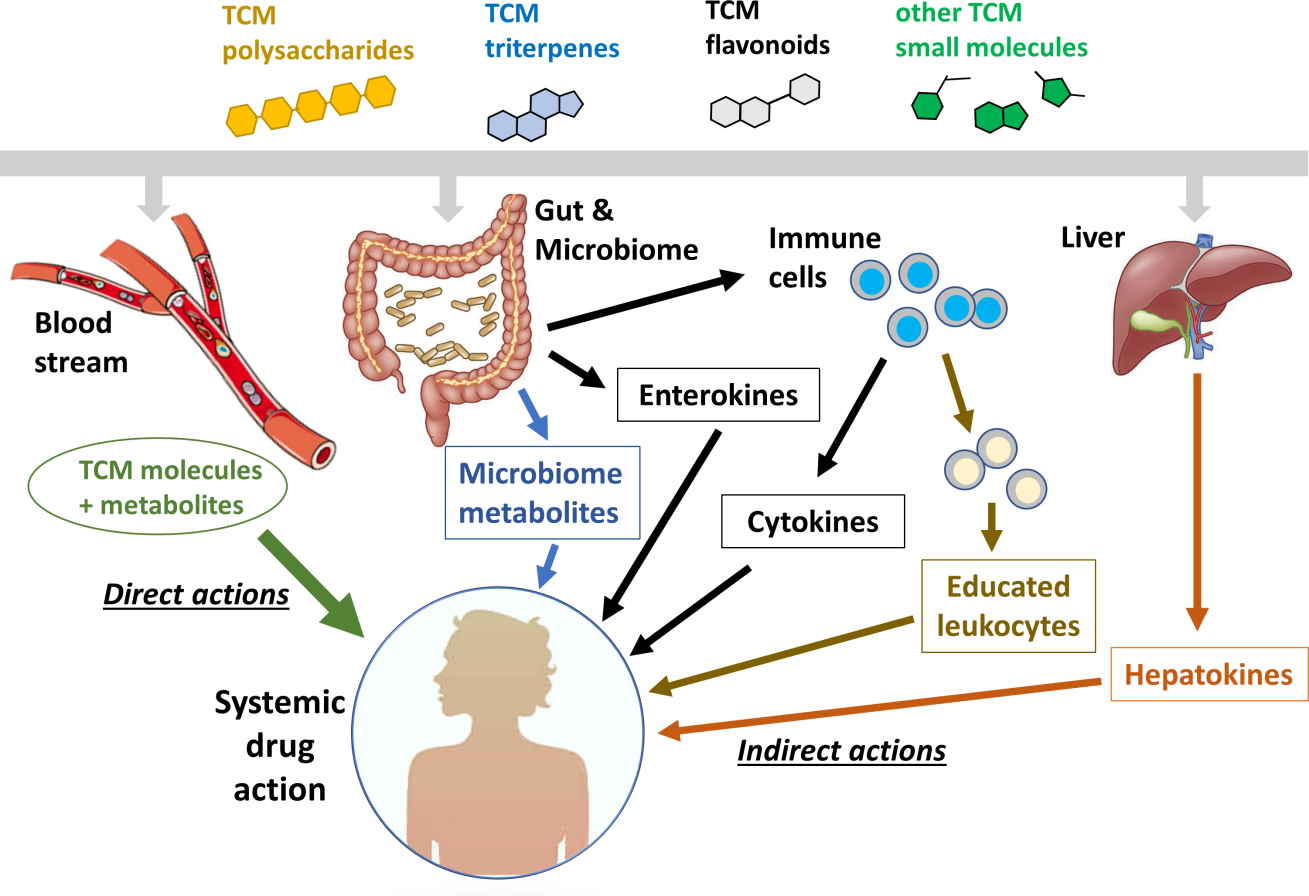
Systemic action of plant- and fungus-derived drugs by direct and indirect mechanisms. This figure compares the conventional view of direct, systemic action (left side) with a number of possible indirect action pathways (right side). The indirect pathways represent mechanisms by which a drug molecule that can only access the gut or liver can achieve systemic activity.

Figure 1 illustrates a series of models to explain how TCM molecules with ‘poor pharmacology’ can act both locally and systemically. The green line on the far left, labeled ‘direct actions’, is the standard oral pharmacology paradigm, where a molecule with ‘good pharmacology’ is absorbed through the gut wall, distributed by the bloodstream and acts directly on target cells throughout the body. Circulating metabolites, usually generated by the liver, may also contribute to systemic action in this model. The other lines illustrate a series of potential indirect action models that may apply to TCM molecules with ‘poor pharmacology’. In these models, the TCM molecule acts locally in the gut or liver to generate mediators that act systemically. These mediators can, in principle, be small molecules, signaling proteins, or immune cells that have been educated by drug exposure or carry insoluble drug particles in their lysosomes. Below, we will explore roles of these indirect mechanisms in the immunomodulatory effects of specific components of traditional herbal medicines. Our emphasis in this review is on pathways in human cells and organs. The blue arrow in Figure 1 indicates direct action of TCM molecules on gut microbes, which is an important research frontier that we do not discuss. For reviews see Lin et al., 2019b; Lin et al., 2021; Zhang et al., 2020a; Li et al., 2021b; Chen et al., 2016.

Before discussing specific exemplars of local pharmacology, we must sound a note of caution. In recent years, TCM has evolved in the direction of injectable formulations that bypass the gut barrier and achieve plasma concentrations that are much higher than those achieved by traditional oral dosing (Li et al., 2018; Jiabo, 2010). For example, an injectable formulation of *Panax ginseng* extract can achieve transient plasma concentrations and systemic exposure (AUC) values of the ginsenoside active ingredients that are many hundreds-fold higher than an oral decoction of the same herb, due to the poor oral bioavailability of ginsenosides (Choi et al., 2020; Zhang et al., 2020b). Injection of TCM ingredients allows for much stronger direct, systemic actions, but at significant risk. The safety of TCM prescriptions is mostly based on experience from traditional oral dosing so it is important to critically evaluate the potential for toxicity when systemic exposure is drastically increased (Li et al., 2019).

## Case studies

Below, we discuss four examples of plant- and fungus-derived molecules with immunomodulatory activity, where we believe restricted distribution and organ-specific pharmacology are central to their therapeutic mechanism. The pharmacology concepts exemplified by these molecules may have much broader implications.

### (1) Indigo – gut-restricted stimulation of AHR by insoluble drug particles

**Figure 2.**
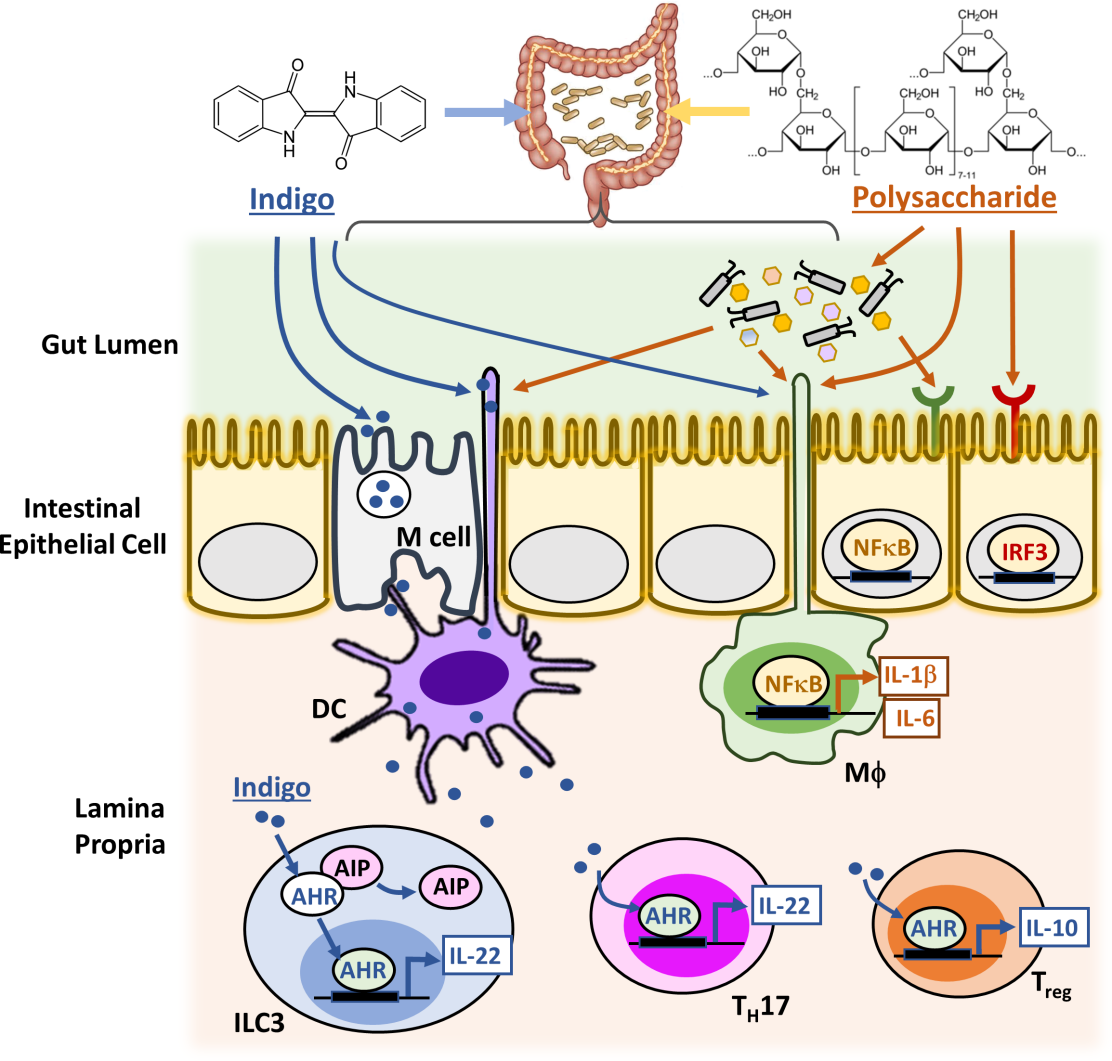
Candidate signaling cascades that mediate the action of Indigo and polysaccharides via the gut. Innate immune cells in the lamina propria, such as macrophages (MΦ) and Dendritic cells (DCs), can continuously sample gut luminal contents, for example, indigo and polysaccharides, by extending the phagocytic processes directly into the gut lumen and also by receiving materials transcytosed out of the lumen by Microfold cells (M cells). This capacity presumably evolved to monitor the gut microbiome and allows the immune system to monitor and respond to molecules that cannot cross the gut wall. Upon activation by xenobiotics and gut microbes, macrophages and DCs upregulate cytokines and chemokines either directly via NFκB or IRF3 signaling pathway, or indirectly by activating other immune cell types in the lamina propria, such as group three innate lymphoid cells (ILC3), T helper 17 (T_H_17) cells and regulatory T (T_reg_) cells. The intestinal epithelial cells (IEC) also perform key innate immune functions with their expressions of distinct TLRs and AHR.

The plant *Indigo naturalis* is best known as a source of blue dye, but it also has traditional medicinal uses in China. Its main bioactive components are the bis-indoles indigo and indirubin, which are formed by non-biological reactions during processing (Sun et al., 2021). Therapeutic benefit of *Indigo naturalis* in ulcerative colitis (UC, a common inflammatory disease of the colon) was proven by recent clinical trials (Naganuma et al., 2018; Naganuma et al., 2020; Uchiyama et al., 2020). Response rates for the herbal medicine were comparable to the most effective western drugs, including biologics that target TNFα, and patients whose disease was not well-managed by western drugs responded well to the herbal preparation (Naganuma et al., 2020). The active ingredients in *Indigo Naturalis*, Indigo and indirubin, are almost insoluble in water and have very poor oral bioavailability (Sun et al., 2021). They are thought to act directly in the gut, where their concentration is locally high following ingestion of *Indigo naturalis* powder. Both are potent agonist ligands of the prototypical xenobiotic sensor AHR (Adachi et al., 2001) and tests in knockout mice showed that AHR expression is required for the therapeutic activity of both *Indigo naturalis* and pure indigo (Kawai et al., 2017). Therapeutic action is thought to proceed via secretion of the protective cytokines IL-10 and IL-22 from gut-associated immune cells (Lin et al., 2019a; Figure 2). The insolubility of indigo makes a traditional oral absorption route unlikely. We propose that indigo particles are delivered to gut macrophages and dendritic cells by phagocytosis, either directly from the gut lumen or following transcytosis by M cells (Bain and Schridde, 2018; Strober, 2009). Once ingested, active molecule slowly leaches from the phagocytosed particle to activate AHR. Limited clinical evidence suggests that the beneficial effects of *Indigo naturalis* power can extend systemically (Sun et al., 2021). This is unlikely to occur by systemic distribution of indigo itself, again due to its insolubility. Rather, it may involve one of the indirect action pathways proposed in Figure 1, such as trafficking of indigo-loaded immune cells from the gut into the systemic immune system. Systemic activation of AHR carries toxicity risk, as exemplified by the reference AHR agonist TCDD-dioxin (Bock and Köhle, 2006). The gut-restricted distribution of the indigo molecule, which we propose is due to its insoluble nature and selective delivery to gut by transcytosis and phagocytosis of particles, may be key to the therapeutic index of *Indigo naturalis* power. One of the proposed functions of AHR in gut macrophages is to monitor the gut microbiome and its metabolites (Stockinger et al., 2021). From this perspective, the therapeutic action of indigo in UC can be viewed as restoring balance to an endogenous immuno-regulatory circuit that monitors the gut microbiome.

### (2) Fungus-derived polysaccharides – protective activation of TLRs in the gut

Fungus-derived polysaccharides are perhaps the most widely studied TCM molecules with immunomodulatory activity. Astragalus, Ganoderma, and Cordyceps are well-known examples of TCM herbs that stimulate the immune system and polysaccharides are believed to be one of their key active components. Lentinan, a polysaccharide derived from shiitake mushroom, was first approved in Japan for treating gastric cancer as a combinatorial agent with standard chemotherapies (Ina et al., 2013). Most of the clinical trial data so far were from Japan and with relatively small patient cohorts (Nakano et al., 1999; Oba et al., 2009; Ina et al., 2011). Although evidence for immunomodulatory effects of TCM-derived polysaccharides is strong, their therapeutic effects in human are in general moderate and they are used mainly as dietary supplements or adjuvant therapies. Available data suggest benefits in prolonging survival and improving the overall wellness of patients with advanced gastric cancer and liver cancer, but large trials with more diverse patient populations are needed to validate efficacy and extend usage. The lack of strong clinical efficacy as defined by Western criteria, such as full and partial tumor regressions, also holds true for other TCM-derived polysaccharides. The challenge and opportunity of developing this class of TCM molecules appear to be not so much in discovering new types of polysaccharides but in designing better clinical trials to evaluate their efficacy as well as benefit to overall patient wellness.

One well-documented immunomodulatory mechanism of TCM-derived polysaccharides is activation of Toll-like Receptors (TLRs) that are expressed on macrophages and dendritic cells. When activated by TCM-derived polysaccharides, they upregulate cytokine signaling. For example, Astragalus polysaccharides were shown to stimulate the TLR4 signaling pathway using a TLR4-defficient mouse model (Zhou et al., 2017). TLRs evolved to detect ‘Pathogen Associated Molecular Patterns’ (PAMPs). Many PAMPs, such as components of bacterial and fungal cell walls, include sugar functionalities that are shared with plant- and fungus-derived TCM molecules, so it makes sense that fungus-derived polysaccharides are TLR activating ligands. The biological effects of TCM-derived polysaccharides are more subtle than classic TLR4 agonists, such as *E. coli* lipopolysaccharide (LPS). Whether this is due to a difference in potency, exposure, or pathways that are induced is not clear. Polysaccharides are probably most active within the gut lumen, where they can interact with the intestinal epithelial cells (IEC), the gut microbiota, specialized immune cells lining the gut and possibly also chemoreceptors in the enteric nervous system (Figure 2). Through symbiotic co-evolution with commensal bacteria, the gut has developed intricate pattern recognition receptor (PRR) signaling cascades in multiple cell types to detect and distinguish different PAMPs so as to maintain homeostasis as well as trigger inflammatory responses (Bain and Schridde, 2018; Strober, 2009; Abreu, 2010). Similar to common PAMPs, activation of intestinal PRR signaling by TCM-derived polysaccharides and/or their metabolites produced by the gut microbes leads to the production of pro-inflammatory cytokines, such as IL-1β, IL-6, and IL-8, and Interferons (IFNα and IFNβ) (Fukata and Arditi, 2013; Kieser and Kagan, 2017). These secondary inflammatory mediators then generate systemic immunomodulatory effects upon secretion to the circulating blood (Schirmer et al., 2016; Mainous et al., 1995). Systemic drug effects may also be mediated by drug-educated immune cells that traffic from the gut to distant sites. For instance, intestinal T cells are known to traffic to extra-intestinal lymph nodes and to the spleen, and recent new data also revealed trafficking of intestinal γδ T cells to the brain that modulates the outcome of acute brain injury (Benakis et al., 2016). In addition, polysaccharide actions on the gut microbiota, for example, which alter the composition and/or metabolic activity of the bacterial species, or the immunogenicity of specific microbes, might also cause changes in the gut and upregulate other systemic immunomodulatory mediators.

### (3) Colchicine - induction of anti-inflammatory hepatokines

**Figure 3.**
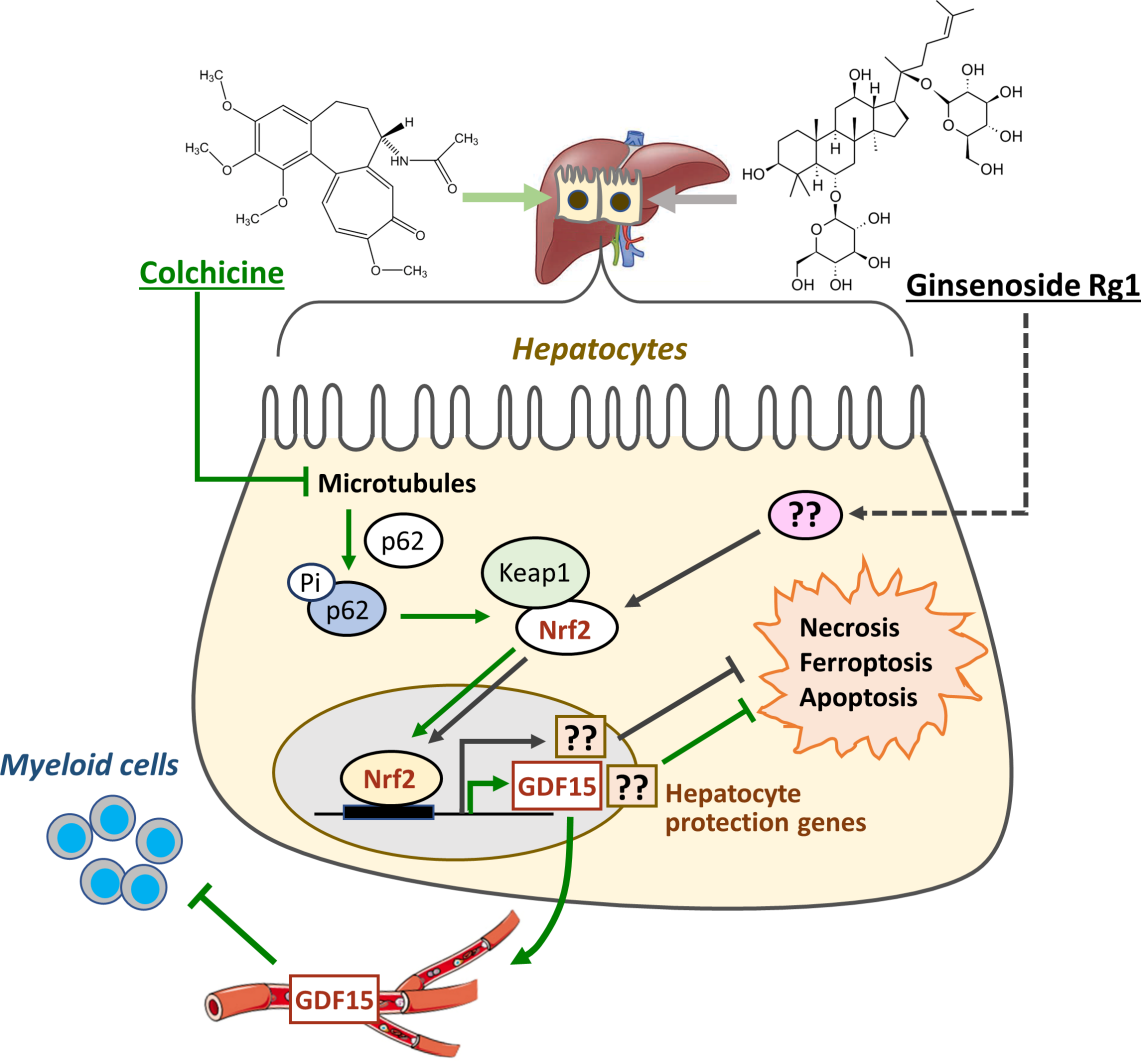
Colchicine and ginsenosides activate protective pathways in the liver. Colchicine inhibits microtubule polymerization selectively in hepatocytes, leading to activation of p62/SQSTM1 and NRF2. NRF2 then induces local cytoprotective proteins as well as a novel anti-inflammatory hepatokine, a form of GDF15, which is secreted into plasma where it acts on circulating myeloid cells. Ginsenosides, which are representative of plant-derived triterpenoids, also activate NRF2 in hepatocytes and induce cytoprotective proteins. Precisely how ginsenosides activate NRF2, and whether they induce hepatokine secretion, are unknown.

The alkaloid colchicine has a long history as a herbal medicine through use of *Colchicum autumnale* in the West and *Gloriosa superba* in Africa and Asia. It is still widely prescribed as a pure molecule for treating gout, Familial Mediterranean Fever and other inflammatory diseases and is being actively explored as a preventive in cardiovascular disease (Dasgeb et al., 2018; Tardif et al., 2019; Cocco et al., 2010). Colchicine targets tubulin, the subunit of microtubules, and promotes their depolymerization (Borisy and Taylor, 1967). Following oral dosing in humans, it reduces the adhesiveness of circulating myeloid cells and inhibits their recruitment to inflamed tissues (Fordham et al., 1981; Malawista, 1968). Colchicine has a high oral bioavailability and the standard model for its systemic anti-inflammatory action proposes that it acts on circulating myeloid cells to inhibit chemotaxis and IL-1β secretion (Dasgeb et al., 2018). However, this model is problematic. Colchicine is rapidly cleared from the blood to the liver (Thomas et al., 1989; Hunter and Klaassen, 1975). This rapid clearance, combined with its very slow association rate constant for tubulin binding, resulted in very low systemic exposure at safe doses, and microtubules in circulating leukocytes were not damaged following a safe, anti-inflammatory dose in mice (Weng et al., 2021). The same is likely true in humans, since colchicine at safe doses lacks anti-mitotic side effects, such as neutropenia and alopecia. In mice, colchicine damaged microtubules selectively in hepatocytes, presumably because it is concentrated across the sinusoidal membrane by xenobiotic transporters (Weng et al., 2021). Microtubule damage in hepatocytes activated NRF2, a master regulator of xenobiotic defenses, leading to induction of cytoprotective defense proteins (Figure 3). NRF2 activation also caused secretion of a novel form of GDF15 that acts systemically to inhibit inflammatory signaling in circulating myeloid cells (Weng et al., 2021). This new, indirect action model for colchicine has not been validated in humans and the anti-inflammatory form of GDF15 produced by the liver requires further characterization. Nevertheless, the concept that a plant-derived molecule with poor pharmacology can systemically modulate the immune system by inducing hepatokines has broad implications for the pharmacology of plant-derived molecules which enter the blood and act in the liver. The signature clinical activity of colchicine is relieving the symptoms of gout, a common rheumatic disease caused by the buildup of urate crystals in joints. Multiple TCM prescriptions are effective for gout treatment (Chi et al., 2020) and one demonstrated equal efficacy and superior therapeutic index when compared to colchicine in a double-blind trial (Wang et al., 2014). It would be interesting to test whether the active molecules in gout-active TCM prescriptions share colchicine’s ability to induce protective hepatokines.

### (4) Ginsenosides – cytoprotective defense induction by a classic TCM molecule

Ginsenosides are a family of triterpenoid glycosides that are among the most studied of all TCM-derived molecules. They are the most abundant bioactive component of *Panax notoginseng*, which has many applications in TCM. Although being prescribed more for vascular diseases than immune disorders, ginsenosides are known to inhibit inflammation and their activity in liver has similarities to colchicine, making them relevant here. It is unclear whether therapeutic benefit of *Panax notoginseng* has been rigorously proven in any specific human disease, but informal evidence abounds. Orally dosed ginsenosides consistently demonstrated hepatoprotective activity in rodent models of liver disease and poisoning (Ning et al., 2018; Gao et al., 2017; Ren et al., 2019), which is consistent with systemic activity. Ginsenosides exhibit poor oral bioavailability, but a small amount enters systemic circulation after oral dosing (Choi et al., 2020). They activate at least two xenobiotic sensor/defense pathways that are prominent in the liver, PXR and NRF2. PXR does not bind ginsenosides directly, but was shown to be necessary for the anti-inflammatory action of Ginsenoside Rb1 in cell culture (Zhang et al., 2015). Ginsenoside Rg1 activates NRF2 in rodent liver following oral dosing, and this activation is required for it to protect the liver from pathology in multiple disease and toxin models (Ning et al., 2018; Gao et al., 2017; Figure 3). Exactly how Rg1 activates NFR2 is unknown, but the data support a model in which ginsenosides, as a class, protect the liver from pathology by inducing xenobiotic defense program via NRF2 and perhaps other xenobiotic sensors, such as PXR. Ginsenosides are non-toxic following parenteral doses that generate much higher plasma concentrations and systemic exposures than oral doses (Choi et al., 2020; Zhang et al., 2020b). This degree of safety at much higher exposure is consistent with their binding to xenobiotic sensors as opposed to other proteins with critical functions in cell and organ survival.

Colchicine and oral ginsenosides both appear to provide therapeutic benefit by activating xenobiotic defenses in the liver, a proposed mechanism that is shared by many other TCM-derived molecules (Li et al., 2021a; Li et al., 2020). Furthermore, many herbal remedies, as well as pure molecules derived from them, are known to cause drug-drug interactions by inducing or inhibiting cytochrome P450s and drug transporters (Pal and Mitra, 2006). From these findings, we can infer that induction of xenobiotic defenses in the liver is likely to be a widespread mechanism by which TCM molecules influence the human body and promote therapeutic benefit. However, the therapeutic benefit and side effects of these TCMs differ greatly and we cannot explain these differences by implicating activation of single defense pathways. One important unknown is how similar liver responses are between different TCM molecules. Liver xenobiotic sensor and defense systems have mostly been investigated from the perspective of drug metabolism and drug-drug interactions (Mackowiak and Wang, 2016; Chai et al., 2016). Their abilities to discriminate between xenobiotics and regulate secretion of different hepatokines are less explored. Systematic analysis of gene expression patterns in rodent liver might help tease out differences between TCMs. A more direct test of the hepatokine induction hypothesis would be to profile immunoregulatory proteins in the plasma of human volunteers following ingestion of TCM prescriptions, which would likely differ between drugs. Another difference between TCMs may be in the extent to which direct action mechanisms, mediated by systemic distribution of a TCM molecule or its metabolites, act in parallel with induced hepatokines or enterokines.

## Strategies to elucidate TCM activities and identify new drug leads

We believe that TCM is a viable starting point in the hunt for new immunomodulators but that previous research was handicapped by assuming systemic exposure, pre-selecting molecules with particular physical properties during fractionation and lack of realistic immune models. Below, we provide a short list of strategies designed to broaden the hunt and discovery of immune-active TCM molecules that were previously overlooked. The TCM field has made huge progress in chemistry and pharmacology in recent decades and powerful strategies for identification of bioactive molecules have been described elsewhere. Therefore, the suggestions below, based on the concepts in Figure 1, are not intended as a universal approach, but rather to supplement current approaches to fractionation and bioactivity analysis.

### (1) Match fractionation methods to formulation

TCMs are typically formulated by boiling dried herbal mixtures in water, or grinding into powders, for oral dosing. They are rarely extracted in alcohol, unlike the ‘tinctures’ that were prevalent for plant-derived medicines in the West. TCM decoctions are often consumed as cloudy soups so they contain both water-soluble and -insoluble molecules. Conventional TCM fractionation efforts usually start with organic solvent-based extraction, which introduces a bias towards smaller and more hydrophobic molecules. This approach rapidly separates hydrophobic small molecules from bulk plant material and succeeds for isolation of molecules with conventionally drug-like properties that are easy to purify using standard chromatography methods. However, it may miss molecules that are not conventionally ‘drug-like’ because they are large, hydrophilic or insoluble. As discussed above (Figure 2), such molecules may be able to act locally in the gut to induce mediators with systemic benefit.

We particularly suspect that water-soluble TCM molecules are under-represented in the literature. We performed a small screen of herbal water extracts and were able to find water-soluble fractions that differentiated and activated monocytes (Peng et al., 2013). Methods for fractionating water-soluble molecules have been improved in recent years due to the strong interest in metabolite profiling (Crutchfield et al., 2010; Lu et al., 2017). These improved chemical biology methods could be applied to purify and characterize the more hydrophilic active components from TCM crude extracts.

### (2) Comprehensive and disease-specific immuno-profiling

**Figure 4.**
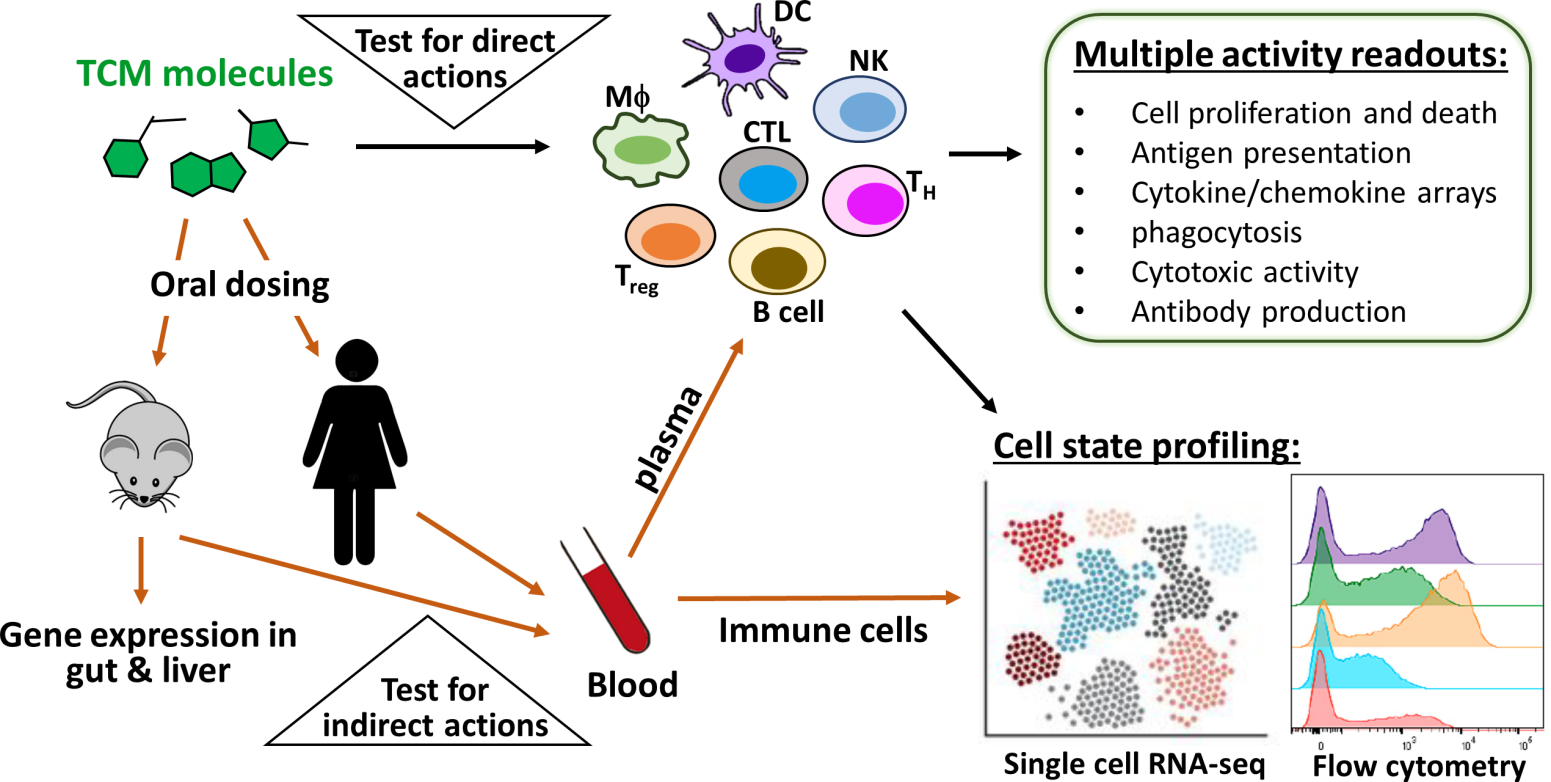
Immuno-profiling of the direct and indirect actions of TCM-derived molecules. To predict direct immunomodulatory activities of TCM-derived molecules, it is necessary to test them in a panel of assays that employ multiple immune cell types, inputs and outputs (upper triangle). To predict indirect immunomodulatory activities, one approach is to orally dose animals or humans, then collect plasma and circulating leukocytes at different time points (lower triangle). The panel of cell-based assays can be re-purposed to discover circulating protein mediators in plasma, such as hepatokines and enterokines. In parallel, flow cytometry and single-cell analysis can be used to detect circulating leukocytes that have been educated by drug exposure. TCM-induced alterations of gene expressions in the gut and liver can also be analyzed from animal tissues to elucidate organ-specific TCM activity.

TCM-derived molecules have often been tested for activity on immune cells in culture, but these tests were typically restricted to one single cell type and measurement. The human immune system is immensely complex and a single measurement is clearly insufficient to predict the immunomodulatory activity of a molecule in vivo. It is now possible to reconstitute many of the cell types and processes that constitute our immune systems in culture, but a comprehensive panel of assays is required (Figure 4). On this point, it is instructive to read papers from pharmaceutical companies which report comprehensive immuno-profiling of small-molecule immunomodulators, such as kinase inhibitors, using a battery of assays with multiple immune cell types, inputs and outputs (Braselmann et al., 2006; Zhu et al., 2007; Blomgren et al., 2020).

While testing across a panel of normal immune cell types is much more informative than a single assay, testing cells only in their normal state may still not be sufficient. Immune cells exhibit specific forms of dis-regulation in disease contexts. If the therapeutic goal is to correct these aberrant cellular phenotypes, it is necessary to model them in culture. For example, there is a need for drugs that rejuvenate exhausted T cells in solid tumors to improve immunotherapy (Jiang et al., 2015). To identify such drug candidates, it is necessary to develop a cell culture model of exhausted T cells and assay their metabolic and cytotoxic activities. The closer the culture model matches the state of T cells in patient tumors, the more predictive the assay will be. Recent development of various single cell analysis methods has revolutionized our ability to profile and describe the variable states of immune cells in disease situations, which, as discussed above, often differ from any known healthy states of the same cells. These data should, in principle, help us model disease-specific states of the immune system in cell culture and then seek molecules that reverse them.

### (3) Screen for protein and cellular mediators in blood

In situations where TCM decoctions act indirectly in the gut or liver to trigger release of systemic mediators (Figure 1), characterization of the circulating mediator is essential for elucidating the overall pharmacology. In the case of colchicine, the key methodological advance was to test plasma from drug-treated mice for immunomodulatory effects in cell-based assays (Weng et al., 2021). This revealed the presence of a colchicine-induced anti-inflammatory hepatokine that was subsequently identified as a form of GDF15 by gene expression profiling of rodent liver. Similarly, a key step in understanding the protective action of indigo in gut inflammation was to measure induction of the anti-inflammatory cytokines IL-10 and IL-22 in gut-associated leukocytes (Lin et al., 2019a). Systemic mediators, whether they be proteins or cells, are accessible in blood, which means that their identification is feasible in human volunteers as well as animal models. The lower triangle in Figure 4 illustrates the relevant approaches.

Measurement of signaling proteins in blood has the benefit of human applicability but will fail to reveal mediators if they are present below detection limits, short-lived or locally acting. For example, the important inflammatory mediators derived from polyunsaturated fatty acids are mostly short-lived, locally acting and require specialized detection methods (Norris and Serhan, 2018). Profiling gene expression in target organs in rodent models is technically easier than profiling proteins in blood. It has often been used to identify drug-modulated pathways in the target organ. We urge scholars to also query secreted proteins that may mediate systemic action of a xenobiotic, especially in the liver given its high capacity to modify blood chemistry via secreted proteins (Shimada and Mitchison, 2019; Weng et al., 2021). Profiling metabolites is another unbiased method that can detect novel pathways that act locally or systemically. Co-culture of tissue cells with immune cells can detect short-lived paracrine signals via their biological effects. This approach was central to the discovery of lipid-derived mediators (Samuelsson et al., 1987) and remains relevant.

### (4) Non-standard TCM prescriptions

Common, standardized TCM prescriptions have been extensively researched and may not be the most productive sources of new drugs. A fascinating, and at times frustrating, aspect of TCM is the existence of multiple parallel schools and traditions, many of them based on regional variation in native flora. The plant, *Alocasia Cucullata* (AC), from which we uncovered a novel water-soluble active fraction with monocyte-differentiating activity (Peng et al., 2013), is mainly used locally in southeast China to treat cancer and infectious diseases, as the plant is native to the mountain areas of southeast China, in particular the Guangdong and Guangxi province. The rapid modernization of China may have led to the loss of some valuable TCM traditions and medicinal species. We therefore suggest that recording the knowledge of local practitioners and increasing the diversity of live botanical resources are urgent.

### Concluding remarks

The principles and practice of TCM and other plant-based medicinal traditions are undergoing rapid modernization in Asia, with improved clinical trials, standardization of ingredients and PK-PD analysis. Convincing reports of beneficial clinical activity from well-executed trials are on the increase. In parallel, informal use of herbal supplements has increased dramatically in the West, with many plant-derived molecules now readily available on the internet. These developments suggest that traditional medicines should be a rich starting point for novel immunomodulatory drugs, yet progress has been slow. One reason may be the necessity for combinations of molecules for clinical activity (Zhang et al., 2014; Li et al., 2012; Ru et al., 2014; Zhao et al., 2020). Here, we argued that another important reason has been the lack of consideration of pharmacology fundamentals. Given the ‘poor pharmacology’ of many plant-derived molecules, local action in the gut and liver may be common, in some cases generating immune mediators that circulate in blood and are responsible for systemic benefit (Figure 1). The discovery that a novel form of the hepatokine GDF15 mediates the systemic anti-inflammatory action of colchicine (so far only in mice) is a recent example (Weng et al., 2021). Also, a focus on molecules that are soluble in organic solvents may have caused chemists to miss valuable immunomodulators that are polar in nature. We hope that the experimental strategies that we discuss, informed by these ideas, provide novel routes to pursue discovery of immunomodulatory drugs from traditional herbal medicines. We strongly believe that these medicines contain molecules with valuable single agent immunomodulatory activities and have the potential to complement both western drugs and the complex decoctions to provide clinical benefit that is broadly applicable and financially affordable.
